# Infections with *Klebsiella pneumoniae* in Children Undergoing Anticancer Therapy or Hematopoietic Cell Transplantation: A Multicenter Nationwide Study

**DOI:** 10.3390/jcm13144078

**Published:** 2024-07-12

**Authors:** Alicja Sękowska, Krzysztof Czyżewski, Kamila Jaremek, Patrycja Zalas-Więcek, Olga Zając-Spychała, Jacek Wachowiak, Anna Szmydki-Baran, Łukasz Hutnik, Agnieszka Gietka, Olga Gryniewicz-Kwiatkowska, Bożenna Dembowska-Bagińska, Katarzyna Semczuk, Katarzyna Dzierżanowska-Fangrat, Wojciech Czogała, Walentyna Balwierz, Iwona Żak, Renata Tomaszewska, Tomasz Szczepański, Ewa Bień, Ninela Irga-Jaworska, Katarzyna Machnik, Justyna Urbańska-Rakus, Sonia Pająk, Marcin Płonowski, Maryna Krawczuk-Rybak, Aleksandra Królak, Tomasz Ociepa, Tomasz Urasiński, Paweł Wawryków, Jarosław Peregud-Pogorzelski, Tomasz Brzeski, Katarzyna Mycko, Hanna Mańko-Glińska, Wanda Badowska, Agnieszka Urbanek-Dądela, Grażyna Karolczyk, Weronika Stolpa, Katarzyna Skowron-Kandzia, Agnieszka Mizia-Malarz, Filip Pierlejewski, Wojciech Młynarski, Jakub Musiał, Radosław Chaber, Joanna Zawitkowska, Agnieszka Zaucha-Prażmo, Katarzyna Drabko, Jolanta Goździk, Jowita Frączkiewicz, Małgorzata Salamonowicz-Bodzioch, Krzysztof Kałwak, Jan Styczyński

**Affiliations:** 1 Microbiology Department, Ludwik Rydygier Collegium Medicum in Bydgoszcz, Nicolaus Copernicus University in Torun, 85-094 Bydgoszcz, Poland; p.zalas@cm.umk.pl; 2 Department of Pediatrics, Hematology and Oncology, Collegium Medicum, Nicolaus Copernicus University in Torun, 85-094 Bydgoszcz, Poland; krzysztofczyzewski@wp.pl (K.C.); kjaremem@gmail.com (K.J.); jstyczynski@cm.umk.pl (J.S.); 3 Department of Pediatric Oncology, Hematology and Transplantology, University of Medical Sciences, 61-701 Poznan, Poland; olga_zajac@wp.pl (O.Z.-S.); wachowiak.jacek@outlook.com (J.W.); 4 Department of Oncology, Pediatric Hematology, Clinical Transplantation and Pediatrics, Medical University of Warsaw, 02-091 Warsaw, Poland; aszmydki@tlen.pl (A.S.-B.); lukasz.hutnik@gmail.com (Ł.H.); 5 Department of Oncology, Children’s Memorial Health Institute, 04-730 Warsaw, Poland; a.gietka@ipczd.pl (A.G.); o.gryniewicz@ipczd.pl (O.G.-K.); b.dembowska@ipczd.pl (B.D.-B.); 6 Department of Clinical Microbiology and Immunology, Children’s Memorial Health Institute, 04-730 Warsaw, Poland; k.semczuk@ipczd.pl (K.S.); k.fangrat@ipczd.pl (K.D.-F.); 7 Department of Pediatric Oncology and Hematology, Institute of Pediatrics, Jagiellonian University Medical College, 31-008 Krakow, Poland; wojciech.czogala@uj.edu.pl (W.C.); balwierz@mp.pl (W.B.); 8 Department of Microbiology, University Children’s Hospital, 30-663 Krakow, Poland; izak@usdk.pl; 9 Department of Pediatric Hematology and Oncology, Silesian Medical University, 41-808 Zabrze, Poland; tomaszewskar@gmail.com (R.T.); szczep57@poczta.onet.pl (T.S.); 10 Department of Pediatrics, Hematology and Oncology, Medical University, 80-210 Gdansk, Poland; ebien@gumed.edu.pl (E.B.); nirga@gumed.edu.pl (N.I.-J.); 11 Division of Pediatric Hematology and Oncology, Chorzow City Hospital, 41-500 Chorzow, Poland; katmachnik@gmail.com (K.M.); justyna.urbanska.rakus@gmail.com (J.U.-R.); s.pajak94@gmail.com (S.P.); 12 Department of Pediatric Oncology and Hematology, Medical University, 15-089 Bialystok, Poland; mar26@mp.pl (M.P.); rybak@umb.edu.pl (M.K.-R.); 13 Department of Pediatrics, Pediatric Hematooncology and Gastroenterology, Pomeranian Medical University, 70-204 Szczecin, Poland; ale.krolak@gmail.com (A.K.); tociepa@gmail.com (T.O.); urasin@sci.pam.szczecin.pl (T.U.); 14 Department of Pediatrics, Pediatric Oncology and Immunology, Pomeranian Medical University, 70-204 Szczecin, Poland; pawel.wawrykow@gmail.com (P.W.); jwperegud@gmail.com (J.P.-P.); 15 Department of Clinical Pediatrics University of Warmia and Mazury in Olsztyn, 10-561 Olsztyn, Poland; tomasz.brzeski@uwm.edu.pl (T.B.); katarzynamycko@tlen.pk (K.M.); haniam@autograf.pl (H.M.-G.); bwan@o2.pl (W.B.); 16 Clinical Divison of Pediatric Oncology and Hematology, Regional Specialised Children’s Hospital in Olsztyn, 10-561 Olsztyn, Poland; 17 Division of Pediatric Hematology and Oncology, Children Hospital, 25-736 Kielce, Poland; agnieszka.urbanek-dadela@wszzkielce.pl (A.U.-D.); grazyna.karolczyk@wszzkielce.pl (G.K.); 18 Division of Pediatric Oncology, Hematology and Chemotherapy, Department of Pediatric, Silesian Medical University, 40-055 Katowice, Poland; wera1pl@poczta.onet.pl (W.S.); kasia.skowron@gmail.com (K.S.-K.); a.mizia@hotmail.com (A.M.-M.); 19 Department of Pediatrics, Hematology and Oncology, Medical University, 90-419 Lodz, Poland; filip.pierlejewski@umed.lodz.pl (F.P.); wojciech.mlynarski@umed.lodz.pl (W.M.); 20 Department of Pediatric Oncohematology, Medical Faculty University of Rzeszow, Clinical Provincial Hospital No. 2, 35-301 Rzeszow, Poland; jakubmusial@gmail.com (J.M.); rchaber@wp.pl (R.C.); 21 Department of Pediatric Hematology, Oncology and Transplantology, Medical University of Lublin, 20-059 Lublin, Poland; jzawitkowska1971@gmail.com (J.Z.); agnieszkazauchaprazmo@umlub.pl (A.Z.-P.); kdrabko@gmail.com (K.D.); 22 Stem Cell Transplant Center, University Children’s Hospital, Department of Clinical Immunology and Transplantology, Jagiellonian University Collegium Medicum, 31-008 Krakow, Poland; jgozdzik@cm-uj.krakow.pl; 23 Department of Pediatric Hematology, Oncology and BMT, Wroclaw Medical University, 50-367 Wrocław, Poland; jowitafr@gmail.com (J.F.); msalamonowicz@poczta.onet.pl (M.S.-B.); krzysztof.kalwak@gmail.com (K.K.)

**Keywords:** acute leukemia, children, hematopoietic cell transplantation, infection, *Klebsiella pneumoniae*

## Abstract

**Background:** *Klebsiella pneumoniae* is a nosocomial pathogen that causes severe infections in immunocompromised patients. The aim of the study was to conduct a microbiological and clinical analysis of *K. pneumoniae* infections in children with malignancies or undergoing hematopoietic cell transplantation in Poland. **Methods:** We conducted a retrospective, multicenter study including children and adolescents under 19 years old treated between 2012 and 2021. We analyzed patients’ characteristics, microbiological data, and the outcomes of antibiotic therapy. **Results**: A total of 9121 newly diagnosed children were treated for malignancy and 1697 pediatric patients underwent hematopoietic cell transplantation. *K. pneumoniae* infections were diagnosed in 527 patients. Their overall incidence was 4.86% in pediatric hematology and oncology patients and 4.95% in patients who underwent hematopoietic cell transplantation. The incidence of infection was higher in patients with acute leukemia than with solid tumors (7.8% vs. 4.1%; OR = 2.0; 95% CI = 1.6–2.4; *p* < 0.0001). The most frequent source of infection was in the urinary tract at 55.2%. More than 57% of *K. pneumoniae* strains were extended-spectrum β-lactamase-positive and almost 34% were multidrug-resistant. Infections with *K. pneumoniae* contributed to death in 3.22% of patients. **Conclusions**: *K. pneumoniae* is one of the most critical pathogens in children suffering from malignancies or undergoing hematopoietic cell transplantation. The incidence of multidrug-resistant *K. pneumoniae* strains is increasing and contributing to poor clinical outcome.

## 1. Introduction

Cancer is the main cause of death worldwide. According to World Health Organization data, every year, 400,000 children suffer from cancer, and almost 100,000 die [1]. Infections are a frequent cause of morbidity and mortality in haematological and oncological children due to a compromised immune system. Moreover, they are one of the most frequent complications reported in children with cancer, and a patient with cancer has a three-times greater risk of dying from a fatal infection than a patient without [2,3,4]. In addition, to a compromised immune system, important risk factors include the following: hospitalization, broad-spectrum antibiotic therapy, invasive procedures, and biomaterials (e.g., catheters, tubes, drains).

*Klebsiella pneumoniae* is one of the most common causes of bacterial infections in children with cancer [4,5,6,7]. It can cause severe infections with high morbidity and mortality, especially in immunocompromised patients [8]. According to the literature, mortality ranges from 13% to 55%, depending on the hospital ward, site of infection, and risk factors [5,9,10]. The highest mortality in *K. pneumoniae* infections is noted in patients with solid tumors, children, and in bloodstream infections. *K. pneumoniae* can cause various infections, but the most common are: bacteriemia (including catheter-related), pneumonia, urinary tract infections, wound infections, and intra-abdominal infections. Moreover, this species often exhibits various mechanisms of antimicrobial resistance (e.g., extended-spectrum β-lactamases—ESβLs, carbapenemases), resulting in limited therapeutic options. Compared to *K. oxytoca*, *K. pneumoniae* strains are isolated several times more often from clinical samples and cause infections more frequently. More importantly, they are also more often multidrug-resistant [11,12,13].

In 2008 Rice [14] proposed the acronym “ESKAPE bugs”, which includes the most dangerous bacteria, not only due to the acquisition of drug resistance, but also because of their virulence. In the acronym, “E” means *Enterococcus faecium*, “S”—*Staphylococcus aureus*, “K”—*K. pneumoniae,* “A”—*Acinetobacter baumannii*, “P”—*Pseudomonas aeruginosa*, and “E”—*Enterobacter* spp. Due to increasing multidrug resistance to antibiotics, *K. pneumoniae* infection is more and more often identified. In 2017, the WHO published the first list of antibiotic-resistant “priority pathogens” that pose the greatest threat to human health. In first priority, defined as critical, next to *A. baumannii* and *P. aeruginosa*, resistant to carbapenems, there are *Enterobacteriaceae* rods resistant to carbapenems and producing ESβLs. According to data from the National Medicine Institute, *K. pneumoniae* is the most frequent Enterobacterales producing carbapenemases in Poland [15]. Based on data from the European Antimicrobial Resistance Surveillance Network [16], the percentage of *K. pneumoniae* isolates in 2022 in Poland resistant to 3rd generation of cephalosporins was 61.9%, to aminoglycosides—47.4%, and to fluoroquinolones—60.6%, making it one of the most resistant bacteria in Europe. The most dangerous situation is observed in the case of carbapenems, where the percentage of resistant strains increased from 8.1% in 2018 to 16.8% in 2022. This situation requires constant monitoring of antibiotic resistance of the most important pathogens, especially those causing infections in people with decreased immunity.

Therefore, the aim of the study was to conduct a microbiological and clinical analysis of *K. pneumoniae* infections in oncology and hematology children in Poland over a 10 year period.

## 2. Materials and Methods

### 2.1. Design of the Study

In this retrospective multicenter nationwide study, we analyzed the epidemiology, risk factors, clinical characteristics, and microbiological features of *K. pneumoniae* infections in pediatric cancer patients or hematopoietic cell transplantation (HCT) recipients.

### 2.2. Patients and Data Collection

We conducted a multicenter study collecting data between 2012 and 2021 in Poland. The data included patients under 19 years who underwent anticancer therapy at 17 Polish pediatric hematology and oncology (PHO) centers or HCT at 6 Polish pediatric centers. Data were collected continuously in 2 year intervals.

### 2.3. Definitions

We evaluated only microbiologically documented *K. pneumoniae* infections. Strains isolated from colonization cases were excluded from the study. The definition of colonization was based on “Gale Encyclopedia Medicine” [17]. *K. pneumoniae* strains were isolated from blood, urine, bronchoalveolar lavage, wound swabs, and tissue samples. Microbiologically documented infections were defined according to the baseline and definitions provided by the Infectious Diseases Working Party of the European Society of Blood and Marrow Transplantation [18]. Bloodstream infections, urinary tract infections, pneumonia, and wound infections were diagnosed by the isolation of bacteria from blood, urine, lower respiratory tract samples, and wound swabs, respectively, along with the presence of clinical symptoms. Infections were comparatively analyzed between patients treated at PHO and HCT centers.

The isolate was classified as multidrug-resistant (MDR) if it demonstrated non-susceptibility to one or more agents in over three antimicrobial categories, and as extensively drug-resistant (XDR) if it was non-susceptible to at least one agent in all, but two or fewer antimicrobial categories [19].

### 2.4. Antimicrobial Prophylaxis

A unified prophylaxis was implemented for oncological patients in the neutropenic phase and those undergoing HCT, with consistent policies across all participating centers.

#### 2.4.1. HCT Patients

For HCT patients in the neutropenic phase or undergoing immunosuppressive therapy, antibacterial prophylaxis primarily consisted of penicillins or cephalosporins, occasionally supplemented with ciprofloxacin (used in daily doses of 2 × 250 mg in patients >12 years who had a documented history of infections with resistant bacteria susceptible for ciprofloxacin). Antifungal prophylaxis protocol included fluconazole (up to 2015), posaconazole, or micafungin, alongside trimethoprim-sulfamethoxazole (TMP/SMX) for *Pneumocystis jiroveci* pneumonia (PCP) prevention. Additionally, antiviral prophylaxis with acyclovir was administrated for up to one year post-HCT.

#### 2.4.2. PHO Patients

All patients diagnosed with malignancy or undergoing immunosuppressive therapy received TMP/SMX against PCP, administered at doses of 4–6 mg TMP/kg/day twice or three times per week. Additionally, all acute myeloblastic leukemia (AML) patients received antiviral and antifungal prophylaxis using with the same compounds as HCT patients. Moreover, most AML patients also received antibiotic prophylaxis as mandated by the international chemotherapy protocol [20]. The choice between empirical or pre-emptive anti-infectious therapy depended on clinical and laboratory symptoms. The preferred initial therapy typically involved ceftriaxone or piperacillin/tazobactam in combination with amikacin, with escalation to carbapenem and vancomycin or linezolid as necessary. Standard environmental prophylaxis protocols were uniformly applied across all centers.

### 2.5. Culture, Identification, and Susceptibility to Antibiotics

Samples were collected in accordance with the conventional procedures for microbiological testing [21]. The isolates were cultured on standard microbiological media, Columbia Blood Agar, MacConkey Agar. The strains were identified by mass spectrometry technique using MALDI TOF MS system (MALDI Biotyper, Bruker Daltonics GmbH&Co, Bremen, Germany). An antimicrobial susceptibility testing was determined using a BD Phoenix™ M50 instrument (Becton-Dickinson, NJ, USA) according to the manufacturer’s instructions. The results of susceptibility to antibiotics were interpreted according to the European Committee on Antimicrobial Susceptibility Testing recommendations [22]. ESβL-type enzyme synthesis was determined using a double disk test.

### 2.6. Statistical Methods

The cumulative incidence of infections was calculated using competing risk analysis, starting from the day of transplantation (HCT setting) or from the day of diagnosis (PHO setting). Infection-free survival was analyzed using the Kaplan-Meier method. Categorical variables were compared using the chi-square test or Fisher’s exact test; non-categorical variables were compared with the Mann–Whitney U test. The analysis of risk factors for the incidence and outcome of *K. pneumoniae* infections was conducted using uni- and multivariate logistic regression models. The following variables were included in the analysis of outcome: age (<10 years vs. >10 years), sex (female vs. male), primary diagnosis (acute leukemia vs. other diagnoses), time to infection (<5 months vs. >5 months), treatment (HCT vs. non-HCT), and duration of anti-*Klebsiella* therapy from the beginning of infection (>15 days vs. ≤15 days). Time to infection was defined as the duration from the day of malignancy diagnosis to the onset of *K. pneumoniae* infection in PHO patients; and from the day of transplantation to the occurence of *K. pneumoniae* infection in HCT patients. Independent analysis was conducted to predict survival following *K. pneumoniae* infection. Odds ratios (ORs) with corresponding 95% confidence intervals (95%CI) were calculated. A *p*-value < 0.05 was considered statistically significant.

## 3. Results

### 3.1. Demographics

Throughout the 10 year study, a total number of 9121 newly diagnosed children were treated for malignancy across 17 PHO centers, while 1697 pediatric patients underwent HCT in 6 transplant centers.

### 3.2. Incidence of Infections

*K. pneumoniae* infections were diagnosed in a total of 527 patients, comprising 443 out of 9121 (4.86%) PHO patients and 84 out of 1697 (4.95%) HCT patients (*p* = 0.8). Among patients diagnosed with *K. pneumoniae* the frequencies of infection were as follows: acute lymphoblastic leukemia (ALL, *n* = 209), central nervous system tumors (CNST, *n* = 65), and acute myeloblastic leukemia (AML, *n* = 59). Detailed data regarding the incidence of *K. pneumoniae* infections are presented in the Table 1.

**Figure 1:**
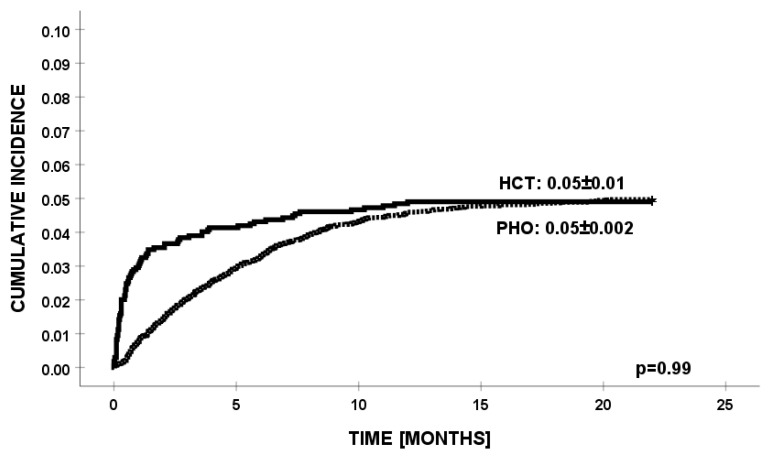
Cumulative incidence of infections with *K. pneumoniae* in HCT and PHO groups.

There was no difference in the cumulative incidence of *K. pneumoniae* infections between PHO and HCT patients (Figure 1).

PHO patients diagnosed with *K. pneumoniae* infection were the most frequently treated for acute lymphoblastic leukemia (ALL, *n* = 176), acute myeloblastic leukemia (AML, *n* = 47), and central nervous system tumors (CNST, *n* = 56). In contrast, in the HCT patients group (*n* = 84), the most common diagnoses were ALL (*n* = 33), AML (*n* = 12), and severe aplastic anemia (SAA, *n* = 9). The most frequent site of infection was the urinary tract in PHO patients (235, 53.0%) and in HCT patients (56, 66.7%). Detailed data regarding patient characteristics and *K. pneumoniae* infections are presented in the Table 2.

The incidence of infection was higher in patients with acute leukemia compared to those with solid tumors (7.8% vs. 4.1%; OR = 2.0; 95%CI = 1.6–2.4; *p* < 0.0001). However, we did not find significant differences between infected and uninfected patients.

### 3.3. Coinfections with Other Pathogens

Species other than *K. pneumoniae* were isolated from 17 (3.2%) clinical samples: 16 (10 urine samples and 6 blood samples) from PHO patients and one from bronchoalveolar lavage from an HCT patient. Alongside *K. pneumoniae*, the most common species were *Escherichia coli* (in seven samples), *Enterobacter cloacae* (in three samples), and *Enterococcus faecalis* (in three samples). The remaining isolates (*Proteus mirabilis*, *Haemophilus influenzae*, *Citrobacter freundii*, *Pseudomonas aeruginosa*, and *Streptococcus oralis*) were cultured from single samples.

### 3.4. Susceptibility to Selected Antibiotics and Mechanisms of Resistance

**Figure 2:**
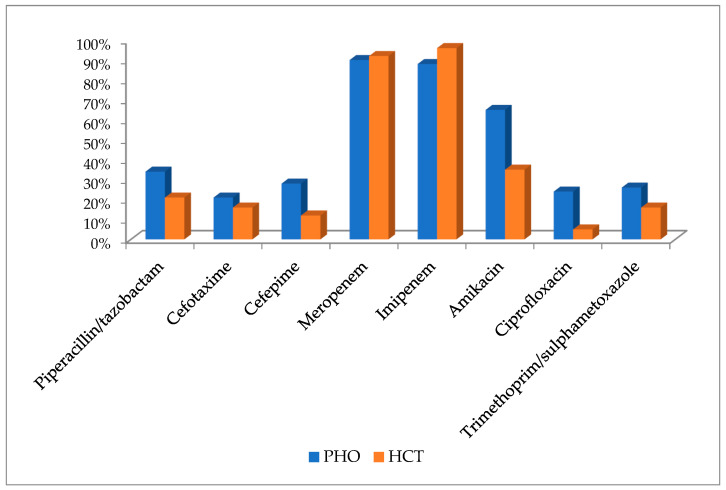
Susceptibility to antibiotics of *K. pneumoniae* strains isolated from PHO and HCT patients.

Susceptibility to selected antibiotics was estimated for 405 (349 isolated from PHO patients and 56—HCT) of *K. pneumoniae* strains. The majority of strains cultured from PHO patients were susceptible to meropenem and imipenem, 315 (90.2%) and 307 (88.0%), respectively. Similarly, the majority of strains cultured from HCT patients were susceptible to imipenem and meropenem, 54 (96.4%) and 52 (92.8%), respectively. In contrast, the lowest numbers and percentages of susceptible strains were observed for cefotaxime—67 strains (19.2%) among PHO patients, and for ciprofloxacin—3 strains (5.4%) among HCT patients. Detailed data regarding susceptibility to selected antibiotics of *K. pneumoniae* strains are presented in the Figure 2.

*K. pneumoniae* ESβL-positive strains were cultured from 301 (57.1%) clinical samples while ESβL-negative strains were found in 226 (42.9%). Out of the 527 isolates, 177 (33.6%) were identified as multidrug-resistant and 10 (1.9%) as extensively drug-resistant. None of the analyzed strains exhibited resistance to all tested antibiotics. Additionally, only six (1.1%) *K. pneumoniae* isolates produced different carbapenemases. Detailed data regarding the phenotypes of the analyzed *K. pneumoniae* strains are presented in Table 3 and Table 4.

### 3.5. Antibiotic Treatment

The most frequently used antibiotics both among 443 PHO and 84 HCT patients were meropenem (45.4% and 39.3%, respectively) and amikacin (27.1% and 28.6%, respectively). Detailed data are presented in Table 5. In 153 (34.5%) PHO patients, combination therapy was applied, most frequently meropenem+amikacin (19 patients), meropenem+vancomycin (16 patients), and piperacillin/tazobactam+amikacin (16 patients). In 32 (38.1%) HCT patients, combination therapy was applied, most frequently meropenem+teicoplanin (6 patients), meropenem+vancomycin (3 patients), meropenem+amikacin (3 patients), and meropenem+colistin (3 patients).

### 3.6. Outcome

Among 527 patients with *K. pneumoniae* infections, in 17 (3.22%) cases an association with death was confirmed. Of these, 12 (70.6%) patients were previously diagnosed with leukemia and 5 (29.4%) patients with solid tumors. In 11 (64.7%) cases, the cause of death was associated with a bloodstream infection. Most *K. pneumoniae* strains isolated from this group of patients were resistant to antibiotics and were classified as MDR or XDR (Table 6).

**Figure 3:**
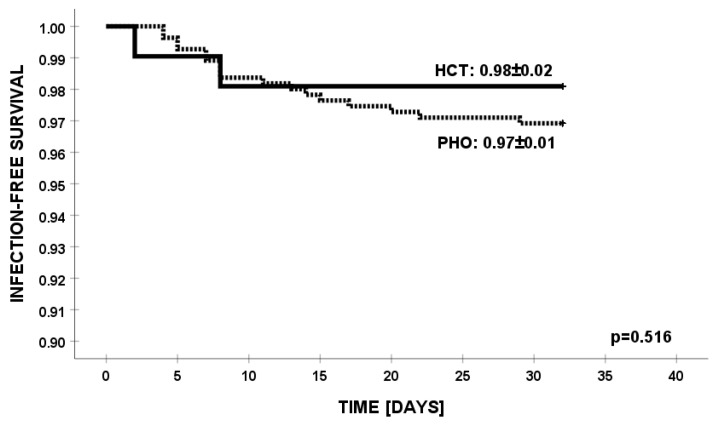
Infection-free survival from infections with *K. pneumoniae* in HCT and PHO groups.

**Figure 4:**
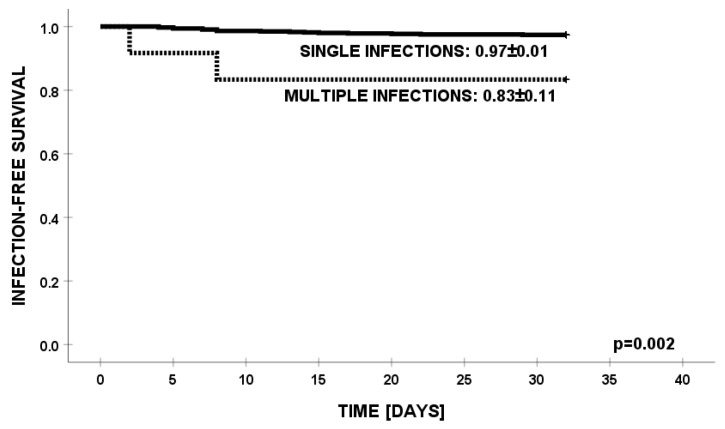
Infection-free survival from infections with *K. pneumoniae* in patients with multiple infections.

There were no differences in the outcomes of therapy for *K. pneumoniae* infections between PHO vs. HCT children (Figure 3). In univariate analysis, we did not find any parameter contributing to the outcome of infections, except for patients infected simultaneously with multiple pathogens (Figure 4).

## 4. Discussion

In this study, we presented the results of a multicenter, nationwide study on the risk factors and outcomes of *K. pneumoniae* infections in children and adolescents following HCT and PHO. Information about *K. pneumoniae* infections in children with malignancies is limited, and mainly concerns adults [6,7,23,24,25,26,27].

In this study, leukemia was the most frequently associated with *K. pneumoniae* infections, accounting for 50.9% of cases. Comparable results were obtained by other authors in pediatric patients [6,7,28,29,30], although the percentages vary significantly, from 28.4% [28] in a study from Colombia to 63.0% in a study from Egypt [7]. In our study, polymicrobial infection was recognized in 3.2% of cases. Similar results were obtained by Lubwama et al. [31] who reported 3.4%, although their study included only 32 strains.

Bacterial infection is one of the most common complications of cancer treatment. The frequency of *K. pneumoniae* infections in patients with cancer varies from 4.6% to 39.0% [31,32]. Al Battashi et al. [30] analyzed infections in children in Oman and found that *K. pneumoniae* was the most frequent pathogen, accounting for 21% of infections, although this study included only nine strains. In turn, Al-Mulla et al. [6], analyzing infections in children under 15 with malignancy, showed that *Staphylococcus epidermidis* was the most common isolated pathogen (22.4%), followed by *K. pneumoniae* (12.1%), making it the most frequent among Gram-negative bacteria. A similar incidence was observed by Joudeh et al. [33], where *K. pneumoniae* (10.0%) was the most frequently isolated Gram-negative pathogen after *Enterococcus faecalis* (18.0%). On the other hand, some authors stated that among Gram-negative bacteria, *K. pneumoniae* is less frequent than *E. coli* [7,32,34,35] with frequency ranging from 6.2% to 37.0%. Conversely, Garg et al. [36], Nirmal et al. [37], and Bhat et al. [25] noted that *Klebsiella* genus was the most frequently isolated pathogen, reaching 47.0%, 29.3% and 18.3%, respectively. However, the authors did not specify what percentages of the strains were *K. pneumoniae*. Interestingly, all three studies were conducted in hospitals in India and cover from one year to four years [36,37]. Moreover, the studies by Nirmal et al. [37] and Bhat et al. [25] focused solely on bloodstream infections, and Garg et al. [36] focused only on patients with cancer hospitalized at Intensive Care Units. Additionally, they included different age groups from 1 to 14 years [37] and from 1 to 72 years [36]. These data demonstrate the multitude of factors that can influence the frequency of isolation of a specific pathogen within a defined group of patients.

In our study, with large cohorts of PHO and HCT pediatric patients, the cumulative incidence of *K. pneumoniae* infections was similar in both settings. This finding emphasizes the comparable level and quality of all prophylactic measures implemented. Additionally, survival after *K. pneumoniae* infections was similar in PHO and HCT patients. This contrasts with data obtained for other Gram-negative pathogens [38], and may reflect similar clinical conditions for both cohorts.

In recent years, although the cancer mortality rate has been decreasing, bacterial infections still remain a significant cause of mortality among patients with malignancy [39]. Mortality depends on various factors associated with the patient (such as age, type of disease, and risk factors) and the microorganism (including species and phenotype) that causes the infection. Amanati et al. [26], in their analysis of adult patients with solid tumors and hematological malignancies, noted a mortality rate of approximately 22% due to *K. pneumoniae* infections, with some differences dependent on factors such as ESβL production and resistance to carbapenems. However, another study by Liu et al. [5] demonstrated a notable disparity in mortality rates between infections caused by carbapenem-susceptible and carbapenem-resistant *K. pneumoniae* strains, of 15.9% and 55%, respectively. In contrast to the aforementioned studies, Al Mulla et al. [6] reported a mortality rate of 10.8% in children with malignancy, with only 2.2% of deaths associated with infection and none attributed to *K. pneumoniae*.

The site of *K. pneumoniae* infection in patients with malignancy may vary, including: urinary tract, lower respiratory tract, skin, and tissue, but bloodstream infections are the most common [27,30,36]. In our study, the most frequent sources of infection were urine, accounting for over 55%, and blood, at almost 42%, in both groups of patients. Similar results were reported by Perdikouri et al. [27] in their analysis of adult patients with malignancy, with rates of 37% and 29%, respectively. In turn, Bhat et al. [25] observed that the frequency of bacterial isolation may be correlated with the type of underlying disease. They found that the lower respiratory tract was the most common site of isolation in patients with solid tumors, while bloodstream infections were more prevalent in those with hematological malignancies. Additionally, Başaran et al. [39] suggested that the site of bacteria isolation might be influenced by the presence of neutropenia. Their research indicated that bloodstream infections were most common in neutropenic patients, whereas urinary tract infections predominated in non-neutropenic patients. In the latter group, bloodstream infections ranked as the fourth most frequent site of isolation. It is important to note that the most cited studies analyzed the isolation of various bacterial species, not only *K. pneumoniae*.

In recent years, there has been observed an increase in the frequency of the isolation of antibiotic-resistant strains with various resistance mechanisms, posing a significant concern in the context of infections complicating the treatment of malignancy. The results of this study revealed that nearly 90% of the analyzed *K. pneumoniae* strains isolated from PHO patients and over 92% from HCT patients were susceptible to carbapenems. Slightly higher susceptibility was reported by researchers from Uganda [31], with over 95%, and from Qatar, where susceptibility reached 100% [6]. In *K. pneumoniae*, the dominant phenotype is ESβL production, although strains producing carbapenemases, MDR, XDR, or PDR, are increasingly being isolated. This study found that over 57% of *K. pneumoniae* strains were ESβL-positive (over 54% in PHO patients and over 71% in HCT patients). In contrast, Amanati et al. [26] analyzed *K. pneumoniae* infections in PHO and HCT patients over a 4 year study and noted ESβL-positive strains ranging from 0% to 33.3% of the total. Conversely, Başaran et al. [39] observed only 1.6% ESβL-positive *K. pneumoniae* strains across all sample types, with 2.5% in blood samples. A higher percentage was reported by researchers from Uganda, with 83% of 32 *K. pneumoniae* isolates being ESβL-positive [31]. The authors from Qatar reported a much lower percentage in PHO children, where only three (21%) of isolates produced ESβL [6]. In this study, low percentages of carbapenemase-positive strains were found, with rates of 1% and 3% for PHO and HCT patients, respectively. Interesingly, the available literature lacks information about carbapenemase-positive *K. pneumoniae* strains, only focusing on carbapenem-resistant strains. A wide range of carbapenem-resistant *K. pneumoniae* strains were reported by Amanati et al. [26] ranging from 0% to 66.7% over a 4 year study period. Conversely, Hattori et al. [40], Erbaş et al. [34], and Chumbita et al. [41] observed low percentages of strains with the aforementioned phenotype, with rates of 0.5%, 1.8%, and 2.0%, respectively. However, it is worth noting that the cited studies included different species of *Klebsiella*, not only *K. pneumoniae*.

In our study, the percentage of *K. pneumoniae* MDR strains ranged from 32% in PHO patients to over 39% in HCT patients. The literature lacks data on strains isolated from children, but in adults, percentages vary widely, reaching from 37.0% to 94.0% in all types of infections [27,31]. Amanati et al. [26] reported a range of *K. pneumoniae* MDR strains from 5% to 17% over their 4 year study period, focusing solely on bloodstream infections. In our study, low percentages were observed for XDR strains, approximately 1% for PHO patients and 6.0% for HCT patients. Notably, only one report, from Uganda [31], described *K. pneumoniae* XDR strains isolated from PHO patients, where the authors identified 56% XDR strains isolated from bloodstream infections, although they analyzed only ESβL-positive strains.

Our study has limitations inherent to its retrospective design. Diagnoses were made at the center level, involving multiple individuals in clinical and microbiological analyses. However, data on the susceptibility of *K. pneumoniae* strains were often limited to selected antibiotics, focusing primarily on resistance mechanisms and determining multidrug-resistance.

In conclusion, it is necessary to recognize *K. pneumoniae* as one of the most common pathogens affecting children with malignancy or undergoing HCT. The frequency of *K. pneumoniae* infections in PHO patients and those undergoing HCT was similar. However, a higher incidence of infection has been observed in children with leukemia compared to those with solid tumors. The results from our study indicate that the frequency of isolation of ESβL-positive *K. pneumoniae* strains is high among Polish PHO and patients undergoing HCT. Moreover, the results suggest a possible association between *K. pneumoniae* infection and mortality, particularly in cases involving MDR or XDR strains. Although carbapenems remain an effective therapeutic option, given the increasing frequency of isolation, special attention should be turned to monitoring XDR and carbapenemase-positive *K. pneumoniae* strains.

## Figures and Tables

**Table 1:** Incidence of *K. pneumoniae* infections with respect to primary diagnosis.

PHO Patients			HCT Patients	
Total	443/9121	(4.86%)	84/1697	(4.95%)
ALL	176/2323	(7.58%)	33/427	(7.73%)
AML	47/432	(10.88%)	12/241	(4.98%)
NHL	35/583	(6.00%)	4/58	(6.90%)
HL	6/675	(0.89%)	1/57	(1.75%)
MDS	1/47	(2.13%)	3/73	(4.11%)
LCH	5/162	(3.09%)		
CNST	56/1512	(3.70%)	9/169	(5.33%)
NBL	43/775	(5.55%)	6/177	(3.39%)
ES	10/158	(6.33%)		
OS	12/109	(11.01%)	6/245	(2.45%)
RMS/STS	23/385	(5.97%)	2/78	(2.56%)
WT	15/521	(2.88%)		
GCT	11/358	(3.07%)		
Other	3/1081	(0.27%)	8/172	(4.65%)

PHO—pediatric hematology and oncology, HCT—hematopoietic cell transplantation, ALL—acute lymphoblastic leukemia; AML—acute myeloblastic leukemia; NHL—non-Hodgkin lymphoma; HL—Hodgkins’ lymphoma; MDS—myelodysplastic syndrome, LCH—Langerhans cell histiocytosis, CNST—central nervous system tumors, NBL—neuroblastoma, ES—Ewing sarcoma, OS—osteosarcoma, RMS—rhabdomyosarcoma; STS—soft tissue sarcoma, WT—Wilms tumor; GCT—germ cell tumor.

**Table 2:** Characteristics of patients infected with *K. pneumoniae*.

PHO Centers (*n* = 443)		HCT Centers (*n* = 84)	
Diagnosis		Diagnosis	
ALL	176 (39.7%)	ALL	33 (39.3%)
AML	47 (10.6%)	AML	12 (14.3%)
NHL	35 (7.9%)	NHL	4 (4.8%)
HL	6 (1.4%)	HL	1 (1.2%)
MDS	1 (0.2%)	MDS	3 (3.6%)
LCH	5 (1.1%)		
CNST	56 (12.6%)	CNST	9 (10.7%)
NBL	43 (9.7%)	NBL	6 (7.1%)
ES	10 (2.3%)		
OS	12 (2.7%)	OS	6 (7.1%)
RMS/STS	23 (5.2%)	RMS/STS	2 (2.4%)
WT	15 (3.4%)		
GCT	11 (2.5%)		
Other	3 (0.7%)	Other	8 (9.5%)
Age (median, min max) [years]	5.8 (0.01–18.0)	Age (median, min max) [years]	9.3 (0.01–19.1)
Gender		Gender	
Girls	191 (43.1%)	Girls	37 (44.0%)
Boys	252 (56.9%)	Boys	47 (56.0%)
Source of infection		Source of infection	
Blood	195 (44.0%)	Blood	24 (28.6%)
Urine	235 (53.0%)	Urine	56 (66.6%)
Wound	8 (1.8%)	Wound	1 (1.2%)
Others	5 (1.2%)	Others	3 (3.6%)

PHO—pediatric hematology and oncology, HCT—hematopoietic cell transplantation, ALL—acute lymphoblastic leukemia; AML—acute myeloblastic leukemia; NHL—non-Hodgkin lymphoma; HL—Hodgkins’ lymphoma; MDS—myelodysplastic syndrome, LCH—Langerhans cell histiocytosis, CNST—central nervous system tumors, NBL neuroblastoma, ES—Ewing sarcoma, OS—osteosarcoma, RMS—rhabdomyosarcoma; STS—soft tissue sarcoma, WT—Wilms tumor; GCT—germ cell tumor.

**Table 3:** Characteristics of *K. pneumoniae* strains isolated from PHO patients.

	ESβL-Positive	ESβL-Negative	MDR	XDR	Carbapenemase-Positive
2020/2021 (*n* = 105)	55 (52.4%)	50 (47.6%)	39	3	3 (KPC, VIM, NDM)
2018/2019 (*n* = 98)	69 (70.4%)	29 (29.6%)	42	1	-
2016/2017 (*n* = 115)	48 (41.7%)	67 (58.3%)	39	1	1 (VIM)
2014/2015 (*n* = 75)	39 (52.0%)	36 (48.0%)	21	-	-
2012/2013 (*n* = 50)	30 (60.0%)	20 (40.0%)	3	-	-
Total (*n* = 443)	241 (54.4%)	202 (45.6%)	144	5	4

ESβL—extended-spectrum β-lactamase, MDR—multidrug-resistant, XDR—extensively drug-resistant, KPC—*Klebsiella pneumoniae* carbapenemase, VIM—Verona integron metallo-β-lactamase, NDM—New Delhi metallo-β-lactamase.

**Table 4:** Characteristics of *K. pneumoniae* strains isolated from HCT patients.

	ESβL-Positive	ESβL-Negative	MDR	XDR	Carbapenemase-Positive
2020/2021 (*n* = 20)	11 (55.0%)	9 (45.0%)	6	3	2 (VIM, NDM)
2018/2019 (*n* = 27)	19 (70.4%)	8 (29.6%)	11	2	1 (VIM)
2016/2017 (*n* = 20)	15 (75.0%)	5 (25.0%)	7	-	-
2014/2015 (*n* = 7)	7 (100.0%)	-	6	-	-
2012/2013 (*n* = 10)	8 (80.0%)	2 (20.0%)	3	-	-
Total (*n* = 84)	60 (71.4%)	24 (28.6%)	33	5	3

ESβL—extended-spectrum β-lactamase, MDR—multidrug-resistant, XDR—extensively drug—resistant, KPC—*Klebsiella pneumoniae* carbapenemase, VIM—Verona integron metallo-β-lactamase, NDM—New Delhi metallo-β-lactamase.

**Table 5:** Antimicrobials used in PHO and HCT patients.

Antimicrobials Used in Therapy	PHO (*n* = 443)	HCT (*n* = 84)
Amoxicillin/clavulanic acid	11 (2.5%)	-
Piperacillin/tazobactam	71 (16.0%)	10 (11.9%)
Cefuroxime	17 (3.8%)	1 (1.2%)
Ceftazidime	33 (7.4%)	6 (7.1%)
Cefotaxime	4 (0.9%)	1 (1.2%)
Ceftriaxone	9 (2.0%)	-
Cefepime	33 (7.4%)	6 (7.1%)
Cefoperazone/sulperazon	14 (3.2%)	-
Ceftazidime/avibactam	-	1 (1.2%)
Meropenem	201 (45.4%)	33 (39.3%)
Imipenem	33 (7.4%)	6 (7.1%)
Ertapenem	6 (1.4%)	-
Gentamicin	3 (0.7%)	-
Amikacin	120 (27.1%)	24 (28.6%)
Ciprofloxacin	20 (4.5%)	3 (3.6%)
Trimethoprim/sulphametoxazole	17 (3.8%)	-
Tigecycline	2 (0.5%)	-
Colistin	6 (1.4%)	6 (7.1%)
Vancomycin	50 (11.3%)	9 (10.7%)
Teicoplanin	16 (3.6%)	12 (14.3%)
Linezolid	13 (2.9%)	2 (2.4%)
Cloxacillin	3 (0.7%)	-
Clarithromycin	1 (0.2%)	-
Azithromycin	1 (0.2%)	-
Clindamycin	1 (0.2%)	2 (2.4%)
No data	18 (4.1%)	15 (17.9%)

**Table 6:** Clinical characteristics of patients who died after *K. pneumoniae* infection.

	HCT/ PHO	Sex	Age (Years)	Disease	Source of Infection	Time from Infection to Death (Days)	Cause of Death	Monomicrobial Infection	ESβL	Carbapenem Resitance	Phenotype of Isolate	Antibiotics Used in Therapy
1	HCT	F	1.7	AML	Blood	8 d	Sepsis	Yes	+	No	MDR	IPM, TEC
2	HCT	F	18.4	AML	Blood	1 d	Bacteremia	Yes	-	No	MDS	MEM, ETP, CIP, CLR, VA, TEC
3	PHO	F	16.3	ALL	Blood	15 d	Progression of maligancy	No *E. coli*	-	No	MDS	FEP, MEM, AN, VA, LZD
4	PHO	F	14.9	ALL	Blood	3 d	Septic shock	Yes	+	No	MDR	MEM, VA, MTR
5	PHO	M	15.6	ALL	Urine	7 d	MOF	Yes	+	No	XDR	IPM
6	PHO	M	15.6	ALL	Blood	20 d	MOF	Yes	+	No	XDR	TZP, MEM
7	PHO	M	3.6	ALL	Urine	19 d	Septic shock	Yes	+	No	MDR	MEM
8	PHO	M	11.6	ALL	Blood	2 d	Septic shock	Yes	+	No	MDR	CAZ, MEM
9	PHO	M	9.6	ALL	Urine	14 d	MOF	Yes	+	IPM-S MEM-R	XDR	MEM, AN, LZD
10	PHO	F	14.8	AML	Blood	1 d	Septic shock	Yes	+	No	MDR	MEM, COL, VA
11	PHO	F	10.1	AML	Blood	8 d	Septic shock	Yes	+	IPM-R MEM-R	XDR	MEM, AN, LZD
12	PHO	F	17.7	AML	Blood	22 d	Sepsis	Yes	-	No	MDS	TZP, MEM, LZD
13	PHO	M	1.3	CNST	Urine	3 d	Progression of maligancy	No *P. mirabilis*	+	No	MDR	IPM
14	PHO	F	12.4	CNST	Blood	5 d	Sepsis	Yes	-	No	MDS	IPM, CIP, MTR
15	PHO	M	1.7	WT	Blood	1 d	Sepsis	Yes	-	No	MDS	MEM, VA
16	PHO	F	17.7	OS	Urine	17 d	Progression of maligancy	Yes	-	No	MDS	TZP, FEP
17	PHO	M	6.4	RMS	Wound swab	29 d	Progression of maligancy	Yes	+	No	MDR	TGC, LZD

AML—acute lymphoblastic leukemia, ALL—acute myeloblastic leukemia, CNST—central nervous system tumor, WT—Wilms tumor, OS—osteosarcoma, RMS—rhabdomyosarcoma, MOF, multi-organ failure, MDS—multidrug-susceptible, MDR—multidrug-resistant, XDR—extensively drug-resistant, TZP—piperacillin-tazobactam, CAZ—ceftazidime, FEP—cefepime, IPM—imipenem, MEM—meropenem, ETP—ertapenem, AN—amikacin, VA—vancomycin, TEC—teicoplanin, LZD—linezolid, CIP—ciprofloxacin, TGC—tigecycline, COL—colistin, CLR—clarithromycin, MTR—metronidazole, IPM-S—imipenem susceptible, IPM-R—imipenem resistant, MEM-R—meropenem resistant.

## Data Availability

The data presented in this study are available on a reasonable request to the corresponding author.
